# CeRNA network reveals potential diagnostic biomarkers or immunotherapy targets for Hypopharyngeal squamous cell carcinoma

**DOI:** 10.1016/j.bjorl.2025.101655

**Published:** 2025-06-25

**Authors:** Xi Yang, Chun Feng, Donghui Jiang, Xin Xu, Yingying Zhang, Jin Wang, Xiaoguang He

**Affiliations:** a The first affiliated Hospital of Kunming Medical University, The Second Department of Otolaryngology, Head and Neck Surgery, Kunming, Yunnan, China; b The Affiliated Hospital of Kunming University of Science and Technology, The First People's Hospital of Yunnan Province, Department of Otolaryngology, Kunming, China; c Kunming Medical University, School of Basic Medical Science, Department of Pathology and Pathophysiology, Kunming, Yunnan, China; d The Affiliated Hospital of Yunnan University, The Second People's Hospital of Yunnan Province, Department of Otolaryngology, Kunming, China

**Keywords:** Hypopharyngeal squamous cell carcinoma, circRNA, ceRNA network, Biomarker

## Abstract

**Objective:**

This study aimed to construct a ceRNA network and identify potential diagnostic biomarkers for HSCC.

**Methods:**

The R software was used to identify Differentially Expressed circular RNAs (DE-circRNAs) and mRNAs (DE-mRNAs) between HSCC and Normal Control (NC) specimens. Univariate Cox analysis and survival curves were used to identify biomarkers associated with the prognosis of HSCC. In addition, the diagnostic value of the biomarkers and their relevance to the tumor microenvironment and immunotherapy were evaluated. A ceRNA network based on mRNAs, circRNAs, and miRNAs was established. Finally, qRT-PCR was used to evaluate the expression of biomarkers in HSCC and para-cancerous tissue samples.

**Results:**

A network containing 90 miRNAs, 47 circRNAs, and 111 mRNAs was established via bioinformatic analysis. Three genes (NRG1, CCNG2, and CHSY1) were identified as prognostic and diagnostic biomarkers for HSCC. The low-CCNG2-expression and low-CHSY1-expression groups had higher survival rates, whereas the low-NRG1-expression group had a lower survival rate. The AUC values of the three biomarkers > 0.9, which indicated that the diagnostic value of all biomarkers was excellent. Moreover, CCNG2 and CHSY1 were associated with the severity of malignancy and the effectiveness of immunotherapy. Subsequently, a ceRNA network containing 3 mRNAs, 7 miRNAs, and 7 circRNAs was established. The results of qRT-PCR validated that the expression patterns of NRG1 and CHSY1 in clinical HSCC and para-cancerous samples were consistent with those observed in the GSE2379 dataset.

**Conclusion:**

In this study, we identified three diagnostic biomarkers for HSCC (NRG1, CCNG2, and CHSY1) and established their corresponding ceRNA network via bioinformatic analysis, providing novel insights into the screening and treatment of HSCC.

## Highlights

• Screened biomarkers related to the HSCC.
• NRG1, CCNG2, CHSY1 with diagnostic value were recognized.
• ceRNA network was established, new insight of HSCC.

## Introduction

Hypopharyngeal Squamous Cell Carcinoma (HSCC) is an aggressive tumour originating from the outer layer (epithelium) of the upper aerodigestive tract^1^ and accounts for 5%–15% of all Head and Neck Squamous Cell Carcinoma (HNSCC).^2^ Due to the unique anatomical location, it is undetectable in the early stages. At present, HSCC is treated with surgical resection combined with adjuvant chemotherapy and radiotherapy in clinical, with a 5-year survival rate of approximately 35%.^3^, ^4^ Therefore, it is critical to identify novel diagnostic and prognostic biomarkers for HSCC and understand the pathological mechanisms underlying its development.

Circular RNA (circRNA) is a kind of special noncoding RNA (noncoding RNA, ncRNA). Most of them are produced by alternative splicing of the precursor mRNA of the protein-encoding gene.^5^ The circRNAs are covalently joined single-strand RNA loops that are abundant in various eukaryotic transcriptomes.^5^ Recent studies have demonstrated that circRNAs regulate the proliferation, invasion, migration, apoptosis, and therapeutic resistance in various human cancers,^6^ including hepatocellular carcinoma,^7^ gastric cancer,^8^ colorectal cancer^9^ and bladder cancer.^10^ Several circRNAs also have been reported to regulate tumorigenesis in HSCC. For example, circMATR3 promotes the proliferation, migration and invasion of HSCC cells.^11^ CircMORC3 is a potential biomarker for early diagnosis of HSCC.^12^ Circ0058106 promotes proliferation, metastasis and epithelial-to-mesenchymal transition EMT process in HSCC.^13^ However, the roles and mechanisms of circRNAs in HSCC remain unclear.

MicroRNAs(miRNAs) are small non-coding RNAs, which can post-transcriptionally regulate target gene expression in tumor progression and metastasis.^14^ miRNAs bind to the 3′-Untranslated Region (UTR) of mRNAs in the cytoplasm to repress translation.^15^ Multiple non-coding RNAs, such as lncRNA, circRNA, small non-coding RNA and pseudogenes, can act as competitive endogenous RNAs (ceRNAs).^16^ CircRNA act as a ‘sponging’ model that absorbs miRNAs and competes with mRNAs.^17^ Studies have shown that abnormal ceRNA networks regulate the development of various cancers,^18^ such as breast, colon, liver, prostate, bladder, lung, gastric and blood tumors. A previous study drew the ceRNA networks of Differentially Expressed Genes (DEGs) in FaDu cell line, in which miR-197-5p and miR-6808-5p might be key genes inducing cisplatin resistance.^19^

In this study, bioinformatic analysis was used to determine the regulatory mechanisms of nc-RNA, construct the ceRNA network, and identify potential diagnostic markers in HSCC. We also obtain the clinical samples of HSCC, and detect the expression of mRNA, which were partially consistent with the results of bioinformatics. Altogether, this study provides valuable insights into the development of HSCC and offers novel diagnostic biomarkers and therapeutic targets for this disease.

## Methods

### Data source

GSE111423 dataset (3 HSCC) specimens and 3 Normal Control (NC) specimens was retrieved from our previous study.^20^ The GSE2379 dataset (GPL91 platform, 14 HSCC specimens and 4 NC specimens) was collected from Gene Expression Omnibus (GEO) database. In addition, the TCGA-HNSC cohort containing the RNA-sequencing (RNA-seq) and survival data of 500 HNSCC and 44 NC specimens was used as the validation dataset.

### Differential expression analysis of GSE111423 and GSE2379 datasets

The differentially expressed circular RNAs (DE-circRNAs) in GSE111423 dataset and differentially expressed mRNAs (DE-mRNAs) in GSE2379 dataset between HSCC and NC specimens were extracted via limma package (v3.42.2) (*p* < 0.05 and |log2FC| > 1).^21^

### Prediction of target miRNAs based on circRNAs and mRNAs

The miRNAs targeting DE-mRNAs were predicted using the miRWalk (http://mirwalk.umm.uni-heidelberg.de/) (score = 0.95, position = 3UTR, miRDB = 1, and TargetScan = 1). The miRNAs targeting DE-circRNAs were mined via circBank database (Tot Score > 200). These two miRNA sets were intersected to obtain common miRNAs. Subsequently, an network among miRNAs, circRNAs, and mRNAs was established via cytoscape software (v3.6.1).^22^

### Functional annotation of mRNAs

The Gene Ontology (GO) and Kyoto Encyclopedia of Genes and Genomes (KEGG) enrichment analysis were implemented via cluster Profiler package (v3.18.0) (adj.p < 0.05).^23^ The enrichment results were presented via ggplot2 package (v3.3.2).^24^

### Identification of prognostic and diagnostic mRNA for HSCC

Univariate Cox regression analysis was utilized to screen for mRNA associated with prognosis in HSCC (HR ≠ 1, *p* < 0.05). The Kaplan-Meier (K-M) curves were painted by survival package (v3.2-7),^25^ The pROC package (v1.18.0) was utilized to paint ROC curves.^26^

### Estimation of immune microenvironment and immunotherapy

Estimate package (v1.0.13) was used to evaluate tumor purity, immune score, stromal score, and ESTIMATE score for HSCC and HNSCC specimens.^27^ The Tumor Immune Dysfunction and Exclusion (TIDE) tool was used to calculate T-cell dysfunction and exclusion scores in patients with HSCC and HNSCC and evaluate their relevance to mRNAs.

### Clinical specimen collection and quantitative reverse transcription PCR

We collected samples of cancer and paracancerous tissue from 10 patients at the first affiliated hospital of Kunming medical University. The diagnosis of HSCC was verified by pathological analysis. This study was approved by the ethics committee of the first affiliated hospital of Kunming medical University. All patients signed an informed consent form before participation. A total of 50 mg of each tissue specimen was homogenized and lysed with TRIzol reagent (Ambion, USA) to extract total RNA. Then, equal amounts of mRNA from the RNA were reverse transcribed into cDNA by SureScript-First-strand-cDNA-synthesis-kit (Servicebio, China). After that, the qPCR was implemented via 2× Universal Blue SYBR Green qPCR Master Mix (Servicebio, China) and CFX Connect real-time quantitative PCR instrument (BIO-RAD, USA). The sequences of the qPCRprimers for each gene and GAPDH were listed in Table 1.

**Table 1:** The primer sequences for q-PCR.

Primer name	Sequence
NRG1 For	GAGGTGAGAACGCCCAAGTC
NRG1 Rev	AAGAAAGCAGCACCAACTGAG
CCNG2 For	CTCCGGCACGATGAAGGATT
CCNG2 Rev	ATCATTCTCCGGGGTAGCCT
CHSY1 For	TCTGGTCTTATGAGATGCAGCAG
CHSY1 Rev	GCAGCTGTATTGTGCGATGG
GAPDH For	CGAAGGTGGAGTCAACGGATTT
GAPDH Rev	ATGGGTGGAATCATATTGGAAC

### Statistical analysis

Publicly accessible bioinformatic databases and *R* software were utilized to analyze and visualize in this study. The heat map was painted using pheatmap package (v1.0.12).^28^ A *p*-value of <0.05 indicated statistically significant differences.

## Results

### Identification of DE-circRNAs and DE-mRNAs in HSCC

**Fig. 1:**
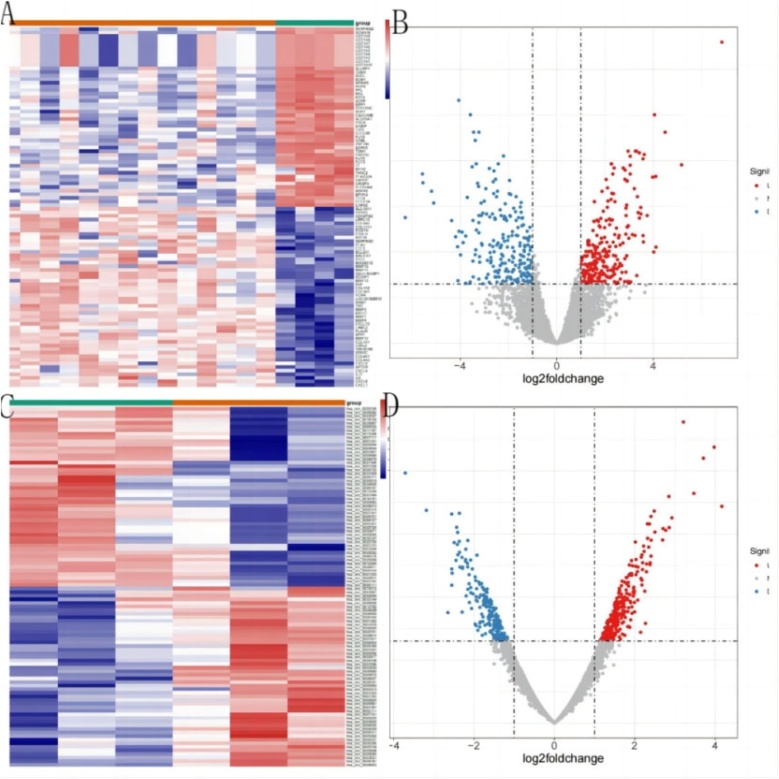
DE-circRNAs between HSCC and NC specimens. (A) 197 circRNAs were downregulated and 265 circRNAs were upregulated in HSCC specimens in the GSE111423197 dataset. (B) Top 50 up- and down-regulated circRNAs. (C) DE-mRNAs between HSCC and NC specimens in the GSE2379 dataset. (D) Top 50 up- and down-regulated mRNAs.

In total, 462 DE-circRNAs were acquired between HSCC and NC specimens in GSE111423, of which 197 circRNAs were lowly expressed and 265 circRNAs were highly expressed in HSCC specimens (Fig. 1A). Fig. 1B displayed the top 50 up- and down-regulated circRNAs. Moreover, 506 DE-mRNAs were recognized between HSCC and NC specimens in GSE2379, of which 267 mRNAs were highly expressed and 239 mRNAs were lowly expressed in HSCC specimens (Fig. 1C). Fig. 1D displayed the top 50 up- and down-regulated mRNAs.

### circRNA-miRNA-mRNA network

**Fig. 2:**
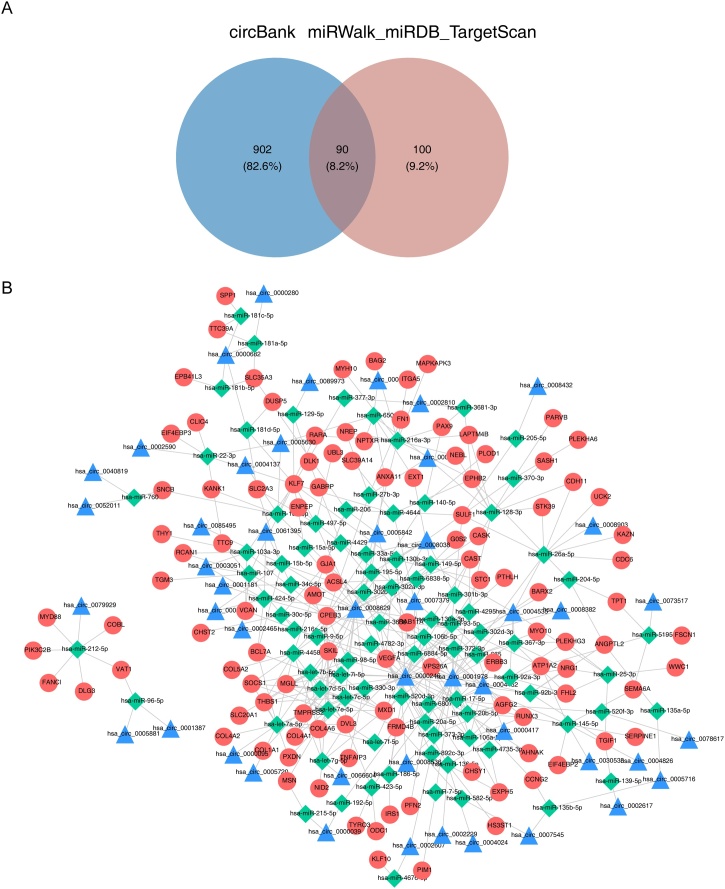
(A) The common miRNAs were acquired by overlapping circBank miRWalk_miRDB_TargetScan. (B) The circRNA-miRNA-mRNA regulatory network.

To investigate the regulatory mechanism of DE-circRNAs and DE-mRNAs, we established the circRNA-miRNA-mRNA regulatory network. A total of 190 miRNAs targeting 142 DE-mRNAs and 992 miRNAs targeting 207 DE-circRNAs were identified. These two miRNA sets were overlapped, resulting in the identification of 90 common miRNAs (Fig. 2A, Table S1). Finally, the network (248 nodes and 445 edges) contained 90 miRNAs, 47 circRNAs, and 111 mRNAs was established (Fig. 2B). Fig. 2B displayed hsa_circ_0000246 and hsa_circ_0001978 could regulate the level of hsa-miR-92b-3p, which in turn affected the expression of NRG1.

### Pathological mechanisms underlying HSCC based on mRNAs

**Fig. 3:**
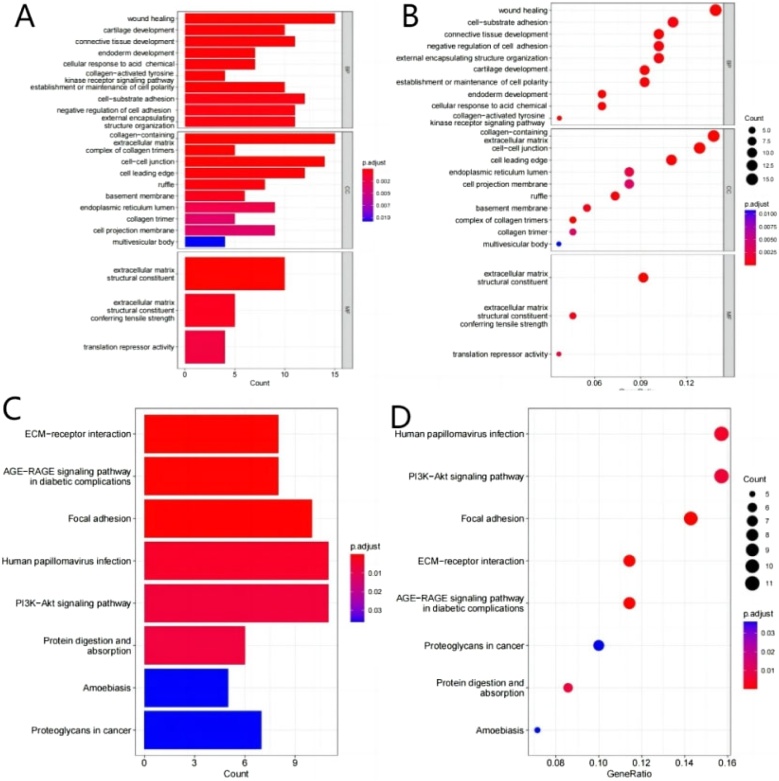
Functional enrichment analysis of 111 mRNAs in the ceRNA network. (A,B) Gene Ontology (GO) analysis showing biological processes and molecular functions. (C,D) Kyoto Encyclopedia of Genes and Genomes (KEGG) pathway analysis.

GO and KEGG enrichment analyses were used to examine the potential biological functions and pathways of 111 mRNAs in the circRNA–miRNA–mRNA network. The GO results demonstrated these mRNAs were associated with the regulation of cell adhesion, response to wounding, establishment or maintenance of cell polarity, and collagen-activated-related signaling pathway (Fig. 3A,B). Moreover, in KEGG terms, mRNAs were associated with human papillomavirus infection and pathways involved in tumorigenesis and progression, such as ECM-receptor interaction, focal adhesion, and PI3K-Akt signaling pathway (Fig. 3C,D).

### Identification of three biomarkers with prognostic and diagnostic value

**Fig. 4:**
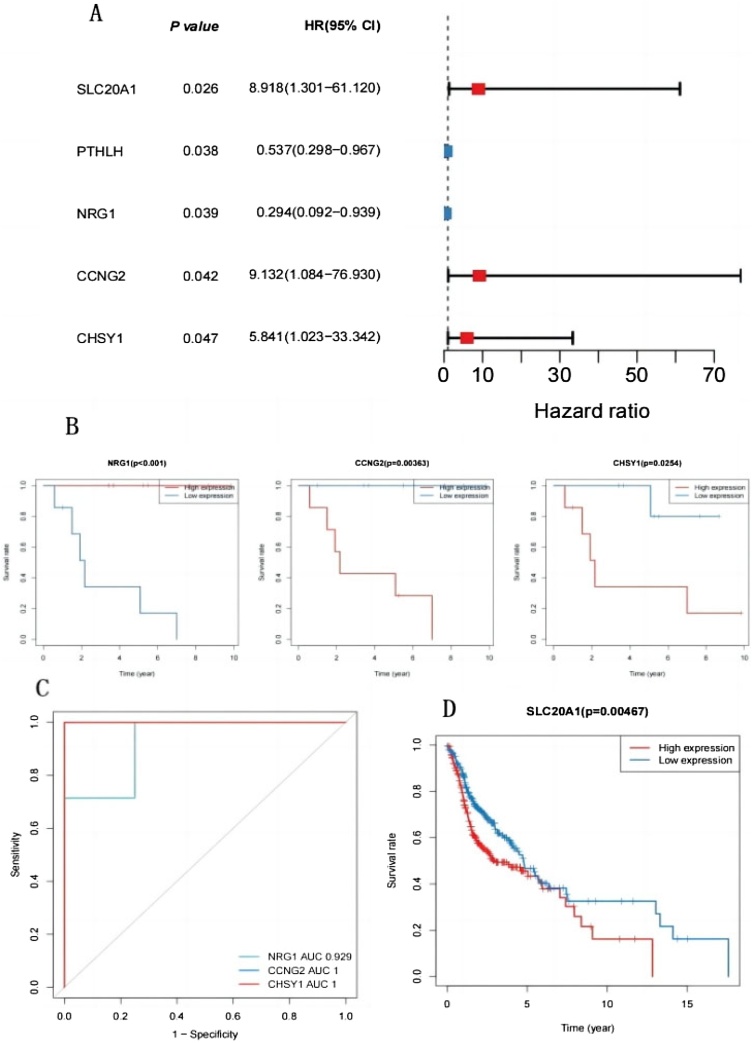
(A) Univariate Cox analysis, 5 mRNAs (SLC20A1, PTHLH, NRG1, CCNG2, and CHSY1) with prognostic value in HSCC. (B) Median expression of 5 mRNAs. K-M curves indicated that 3 mRNAs (NRG1, CCNG2, and CHSY1) were further screened out via *p* < 0.05. (C) The AUC value of 3 mRNAs was all greater than 0.9. (D) The survival rate was notably different between the high- and low-expression groups of SLC20A1, which validated the prognostic value of 5 mRNAs in TCGA-HNSC dataset.

The expression profiles of 111 mRNAs in the circRNA–miRNA–mRNA network were assessed via univariate Cox analysis. In total, 5 mRNAs (SLC20A1, PTHLH, NRG1, CCNG2, and CHSY1) with prognostic value for HSCC were tapped (Fig. 4A). Subsequently, HSCC specimens were divided into high- and low-expression groups based on the median expression levels of the 5 mRNAs. K-M curves showed that NRG1, CCNG2, and CHSY1 had a *p*-value of <0.05 (Fig. 4B). The low-CCNG2-expression and low-CHSY1-expression groups had higher survival rates than their corresponding high-expression groups, whereas the low-NRG1-expression group had a lower survival rate than its corresponding high-expression group. The AUC values of the 3 mRNAs exceeded 0.9, which highlighted the diagnostic potential of these genes in HSCC (Fig. 4C). Therefore, the 3 mRNAs were identified as potential biomarkers for HSCC. Furthermore, we validated the prognostic value of the 5 mRNAs in the TCGA-HNSC dataset. Patients with HNSCC in this dataset were divided into high- and low-expression groups based on the median expression levels of the 5 mRNAs. As shown in Fig. 4D, the survival rate was notably lower in the high-SLC20A1-expression group than in the low-SLC20A1-expression group in the TCGA-HNSC dataset.

### Biomarkers predicted outcomes of immunotherapy in HSCC patients

**Fig. 5:**
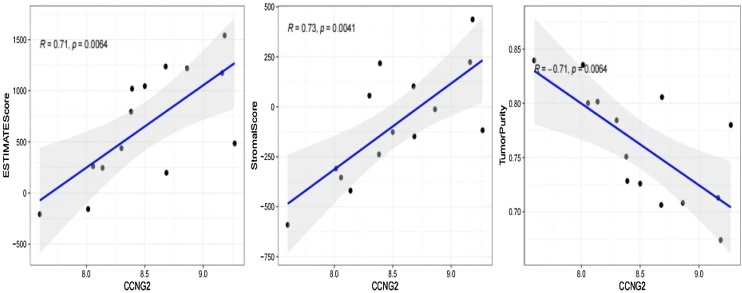
CCNG2 was positively associated with stromal score and ESTIMATE score, and was negatively associated with tumor purity in HSCC.

**Fig. 6:**
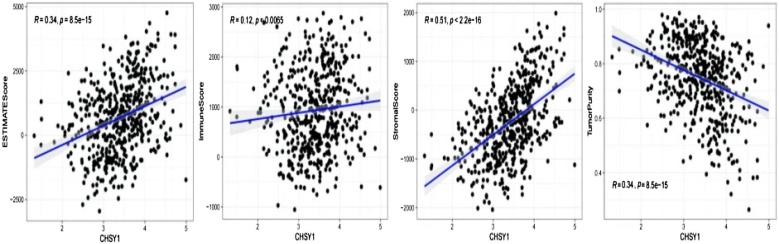
CHSY1 was positively associated with stromal score and ESTIMATE score, and was negatively associated with tumor purity in HNSCC (|cor| > 0.3 and *p* < 0.05).

**Fig. 7:**
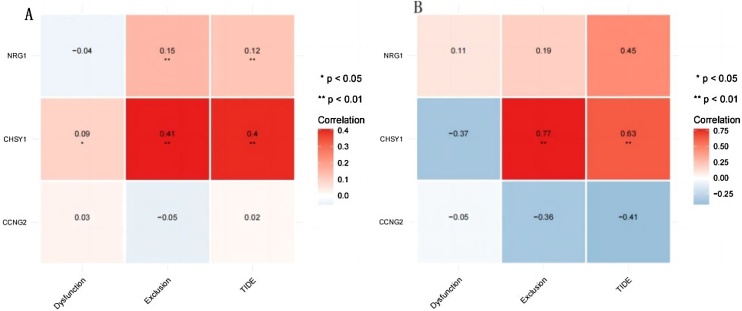
(A,B) TIDE score indicating that as the expression of CHSY1 reduced the immunotherapeutic effect in HSCC and HNSCC patients.

Given that immunotherapy is effective in treating cancer, we investigated the relationship between the identified biomarkers and immune infiltration/immunotherapeutic efficacy. Fig. 5 revealed CCNG2 was notably positively associated with stromal score and ESTIMATE score, and was negatively associated with tumor purity in HSCC. Meanwhile, Fig. 6 revealed that CHSY1 was notably positively associated with stromal score and ESTIMATE score, and was negatively associated with tumor purity in HNSCC (|cor| > 0.3 and *p* < 0.05). Furthermore, the relevance of biomarkers to immunotherapy response was delved in HSCC and HNSCC patients. The result demonstrated CHSY1 was notably positively associated with TIDE score, indicating that an increase in CHSY1 expression attenuated the effects of immunotherapy (Figs. 7A,B). Altogether, above results indicated CCNG2 and CHSY1 were associated with the malignancy of tumor and the effectiveness of immunotherapy.

### Establishment of ceRNA network for biomarkers

**Fig. 8:**
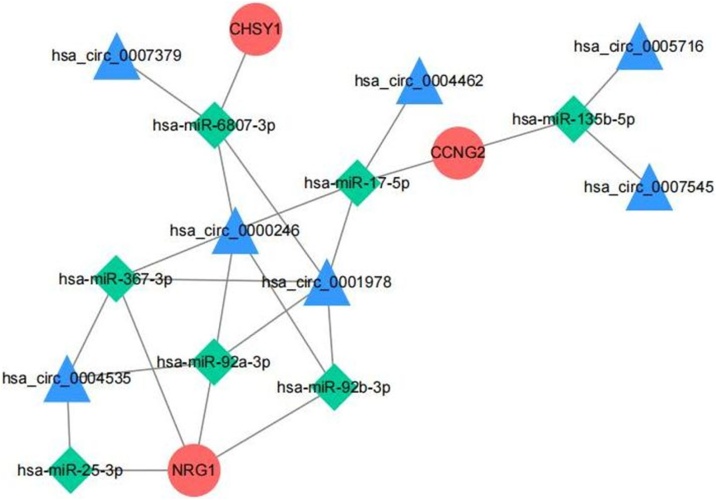
ceRNA network of circRNAs, miRNAs, and mRNAs.

To investigate the regulatory mechanism of biomarkers, we established the ceRNA network depending on the circRNA-miRNA-mRNA network. The ceRNA network (17 nodes and 24 edges) contained 3 mRNAs, 7 miRNAs, and 7 circRNAs. Fig. 8 revealed hsa_circ_0000246 and hsa_circ_0001978 could regulate the level of hsa-miR-6807-3p and hsa-miR-17-5p, which in turn affected the expression of CHSY1 and CCNG2.

### The expression of biomarkers in HSCC

**Fig. 9:**
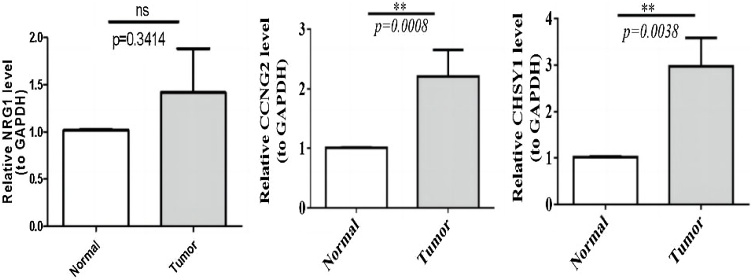
Expression of biomarkers at the 3 mRNA (NRG1, CCNG2, CHSY1) in clinical HSCC samples.

As shown in Table 2, CHSY1 and NRG1 were up-regulated, while CCNG2 was down-regulated in HSCC tissues relative to normal tissues. We used qRT-PCR to validate the mRNA expression levels of these three biomarkers in clinical HSCC and para-cancerous samples. Consistent with the results of the bioinformatic analysis, the mRNA expression levels of CHSY1 and NRG1 were significantly higher in HSCC samples than in para-cancerous samples (Fig. 9). However, the mRNA expression patterns of CCNG2 in clinical samples were opposite to those observed in the GEO dataset (Fig. 9). This discrepancy may be attributed to sample heterogeneity or differences in experimental conditions, highlighting the requirement for further validation of the findings in larger cohorts.

**Table 2:** The detailed information of differential expression of three biomarkers in GSE2379 dataset.

Gene	logFC	AveExpr	*t*	*p*-value	adj.p-val	B
CHSY1	1.289435	9.174195	4.82825	0.000109	0.006257	1.299072
CCNG2	−1.19651	8.781956	−4.26847	0.000393	0.014668	0.058627
NRG1	1.699521	7.999207	3.637496	0.00169	0.037402	−1.33863

## Discussion

HSCC has progressive behavior and poor prognosis, and most patients die owing to metastasis.^29^ Despite the recent improvements in modern comprehensive treatment strategies, including surgery, chemotherapy and radiotherapy. However, survival rates of HSCC has not markedly improved. In this study, we used datasets from GEO and TCGA database to investigate the regulatory mechanism of DE-RNAs, and identify the biomarkers with prognostic and diagnostic value in HSCC. We also established the circRNA-miRNA-mRNA network, which is involved in the tumorigenesis and progression.

The ceRNA network developed in this study contained 90 miRNAs, 47 circRNAs, and 111 mRNAs obtained based on the RNA-seq data of patients with HSCC. GO and KEGG analyses were used to determine the pathways and functions 0f 111 mRNAs were involved. The results of biological processes under GO suggested that specific genes may be concentrated in several process areas, such as cell adhesion, response to wounding, ECM-receptor interaction, focal adhesion, and PI3K-Akt signaling pathway. Interactions of the Extracellular Matrix (ECM) and cellular receptors constitute one of the crucial pathways involved in colorectal cancer progression and metastasis.^30^ PI3K-Akt signaling is involved in cell proliferation and growth as well as down-regulating cell apoptosis.^31^ In previous studies, some of the annotated pathways also have been shown to be associated with cancer, such as breast cancer,^32^ and nasopharyngeal carcinoma.^33^, ^34^ Therefore, we speculate that the above pathways and functions may play an important role in HSCC development through specific mRNAs in the ceRNA network.

Survival analysis revealed 3 mRNAs with prognostic and diagnostic value in HSCC, namely, NRG1, CCNG2, and CHSY1. These mRNAs are promising diagnostic and prognostic biomarkers for HSCC. Further analysis showed that CCNG2 and CHSY1 were significantly associated with the tumor immune microenvironment. In particular, CCNG2 and CHSY1 may be involved in regulating immune infiltration in HSCC tissues. Therefore, these genes are promising immunotherapy targets for HSCC.

Neuregulin 1 (NRG1) gene fusion was detected in various carcinomas. It is most frequently found in Lung Adenocarcinomas (LUAD), especially in KRAS and BRAF wild-type cases.^33^, ^34^ NRG1 is an oncogene that has attracted increased interest in recent years and serves as a potential therapeutic target.^35^ The cyclin family protein CCNG2 acts as a tumor suppressor gene through its regulation of cell proliferation. Low expression of CCNG2 was correlated with the severity of astrocytoma.^36^ Abnormal expression of CCNG2 had been examined in esophageal cancer,^37^ and nasopharyngeal carcinoma.^38^ The carcinogenic effects of Chondroitin sulfate synthase 1 (CHSY1) have been reported in a variety of human cancers, such as gastric cancer,^39^ colorectal cancer,^40^ and hepatocellular carcinoma.^41^ However, these 3 mRNAs have not been reported in previous studies of HSCC. Therefore, these mRNAs are novel diagnostic biomarkers and immunotherapy targets for HSCC.

In our study, hsa_circ_0000246/hsa_circ_0001978/hsa-miR-17-5p/CCNG2 axis is a key ceRNA network in HSCC. Li et al. demonstrated that miR-17-5p is highly expressed in LUAD and is associated with patient prognosis as a novel marker for clinical diagnosis of Non-Small-Cell Lung Cancer (NSCLC).^42^, ^43^ To date, no studies have investigated the the role of hsa_circ_0001978 and hsa_circ_0000246 in cancer. The opposite expression patterns of CCNG2 in clinical samples and the GEO dataset may be due to sample heterogeneity. Therefore, data from a larger range of clinical samples are needed to further validate.

Although we identified several potential diagnostic and prognostic biomarkers and a ceRNA network in HSCC, the results were mainly based on bioinformatic analysis. The relatively small size of clinical specimens limits the statistical power and generalizability of the findings. Therefore, studies with larger cohorts are warranted to validate the findings. In future studies, we will design cellular or animal experiments to verify the biological functions of the three biomarkers and the specific mechanism of the ceRNA network.

## Conclusions

In this study, we identified diagnostic and prognostic biomarkers for HSCC using public databases and bioinformatic methods. The role of these biomarkers and their relationship with HSCC warrant further investigation. In conclusion, the biomarkers and ceRNA network proposed in this study provide novel insights into the diagnosis and treatment of HSCC.

## Meeting of ethical standards

The investigation conformed to the principles outlined in the Declaration of Helsinki, and written informed consent was obtained from all participants. The Ethics Committee of the first affiliated hospital of Kunming medical University approved this study.

## Funding

This study was supported by Yunnan Provincial Department of Science and Technology-Kunming Medical University Joint Project on Applied Basic Research (202301AY070001-208).

## Declaration of competing interest

The authors declare no conflicts of interest.
