# Supplementary material for: CeRNA network reveals potential diagnostic biomarkers or immunotherapy targets for Hypopharyngeal squamous cell carcinoma
Source: Braz J Otorhinolaryngol. 2025 Jun 25;91(5):101655. doi: 10.1016/j.bjorl.2025.101655 (PMC12246853; doi:10.1016/j.bjorl.2025.101655)
Supplement: Supplementary file 1 [file mmc1.docx]

BJORL-D-24-00421_Supplementary Material

**Supplementary Table 1** 90 common miRNAs targeting DE-mRNAs and DE-circRNAs.

| hsa | let | 7d | 5p |
| --- | --- | --- | --- |
| hsa | miR | 149 | 5p |
| hsa | miR | 6504 | 5p |
| hsa | miR | 30c | 5p |
| hsa | miR | 206 |  |
| hsa | miR | 3666 |  |
| hsa | miR | 140 | 5p |
| hsa | miR | 4458 |  |
| hsa | miR | 181c | 5p |
| hsa | miR | 145 | 5p |
| hsa | miR | 17 | 5p |
| hsa | miR | 93 | 5p |
| hsa | miR | 5195 | 3p |
| hsa | miR | 216a | 3p |
| hsa | miR | 138 | 5p |
| hsa | miR | 760 |  |
| hsa | let | 7a | 5p |
| hsa | let | 7b | 5p |
| hsa | let | 7c | 5p |
| hsa | miR | 98 | 5p |
| hsa | let | 7i | 5p |
| hsa | let | 7e | 5p |
| hsa | miR | 370 | 3p |
| hsa | miR | 106b | 5p |
| hsa | miR | 20b | 5p |
| hsa | miR | 26a | 5p |
| hsa | miR | 424 | 5p |
| hsa | miR | 15b | 5p |
| hsa | miR | 302a | 3p |
| hsa | miR | 302b | 3p |
| hsa | miR | 520d | 3p |
| hsa | miR | 892c | 3p |
| hsa | miR | 195 | 5p |
| hsa | miR | 20a | 5p |
| hsa | miR | 106a | 5p |
| hsa | miR | 4676 | 3p |
| hsa | miR | 25 | 3p |
| hsa | miR | 367 | 3p |
| hsa | miR | 92a | 3p |
| hsa | miR | 92b | 3p |
| hsa | miR | 130a | 3p |
| hsa | miR | 301b | 3p |
| hsa | miR | 4295 |  |
| hsa | miR | 6838 | 5p |
| hsa | miR | 130b | 3p |
| hsa | miR | 181a | 5p |
| hsa | miR | 212 | 5p |
| hsa | miR | 107 |  |
| hsa | miR | 103a | 3p |
| hsa | miR | 9 | 5p |
| hsa | miR | 205 | 5p |
| hsa | miR | 4644 |  |
| hsa | let | 7g | 5p |
| hsa | miR | 27b | 3p |
| hsa | miR | 136 | 5p |
| hsa | miR | 4735 | 3p |
| hsa | miR | 128 | 3p |
| hsa | miR | 33a | 5p |
| hsa | miR | 4782 | 3p |
| hsa | miR | 6884 | 5p |
| hsa | miR | 302d | 3p |
| hsa | miR | 582 | 5p |
| hsa | miR | 372 | 3p |
| hsa | miR | 4429 |  |
| hsa | miR | 7 | 5p |
| hsa | miR | 330 | 3p |
| hsa | miR | 15a | 5p |
| hsa | miR | 497 | 5p |
| hsa | miR | 181d | 5p |
| hsa | miR | 22 | 3p |
| hsa | miR | 129 | 5p |
| hsa | miR | 377 | 3p |
| hsa | miR | 181b | 5p |
| hsa | miR | 139 | 5p |
| hsa | miR | 204 | 5p |
| hsa | miR | 423 | 5p |
| hsa | let | 7f | 5p |
| hsa | miR | 3681 | 3p |
| hsa | miR | 34c | 5p |
| hsa | miR | 215 | 5p |
| hsa | miR | 135b | 5p |
| hsa | miR | 186 | 5p |
| hsa | miR | 520f | 3p |
| hsa | miR | 373 | 3p |
| hsa | miR | 96 | 5p |
| hsa | miR | 135a | 5p |
| hsa | miR | 665 |  |
| hsa | miR | 6807 | 3p |
| hsa | miR | 192 | 5p |
| hsa | miR | 216a | 5p |
